# Effects of Corn Silage Inclusion Level and Type of Anabolic Implant on Animal Growth Performance, Apparent Total Tract Digestibility, Beef Production per Hectare, and Carcass Characteristics of Finishing Steers

**DOI:** 10.3390/ani11020579

**Published:** 2021-02-23

**Authors:** Elizabeth M. Buckhaus, Zachary K. Smith

**Affiliations:** Department of Animal Science, South Dakota State University, Brookings, SD 57007, USA; elizabeth.buckhaus@sdstate.edu

**Keywords:** feedlot, growth, integrated systems, apparent total tract digestibility

## Abstract

**Simple Summary:**

Corn silage has long been a staple feed ingredient in cattle diets throughout the Midwest region of the United States. The most recent Feedlot Consulting Nutritionist Survey indicated that corn silage was the primary and secondary roughage source used in finishing diets by 37.5% of respondents. Using the entirety of the corn plant for feed allows for producers to quickly harvest feed tonnage. A common belief is that one should only include enough corn silage in a finishing ration to maintain rumen health. However, previous research conducted by our research group suggested that feeding increased levels of corn silage could increase the quantity of beef produced per hectare of cropland. Another aspect to consider when finishing cattle is implant type. Today a wide variety of implants are available, including polymer barrier coated and non-coated implants. Coated implants can provide an extended release of trenbolone acetate and estradiol for up to 200 days post-implantation. The objectives of the current study were to determine the influence of corn silage inclusion level and terminal implant type on animal growth performance, apparent total tract digestibility, carcass traits, and beef production per hectare of cropland in finishing steers harvested at common rib fat thickness.

**Abstract:**

Maine-Anjou × Angus cross-bred steers (*n* = 156 steers; initial body weight (BW) 366 ± 37.2 kg) were used in a 132 d finishing study conducted at the Ruminant Nutrition Center (RNC) in Brookings, SD. Steers were blocked by weight (*n* = 5 BW blocks) and randomly assigned to an implant and dietary treatment of a randomized complete block design with each pen containing seven to eight steers (*n* = 20 pens). Dietary treatments consisted of (1) 15% (CS15) or (2) 30% corn silage (CS30) where corn silage displaced corn grain in the diet. Steers received one of two implants (both from Zoetis, Parsippany, NJ) containing equal doses of trenbolone acetate (TBA) and estradiol benzoate (EB): (1) Synovex PLUS (non-coated implant; 200 mg TBA and 28 mg EB; PLUS) or (2) Synovex ONE Feedlot (coated implant; 200 mg TBA and 28 mg EB; ONE-F). Bunks were managed using a slick bunk approach, and all diets contained dry matter (DM) basis 33 mg/kg monensin sodium. All steers were offered *ad libitum* access to feed, and feeding occurred twice daily in equal portions. There was no interaction between the implant and dietary treatment for any variables measured (*p* ≥ 0.08). Carcass-adjusted basis final BW, average daily gain (ADG), and grain to feed (G:F) were increased (*p* ≤ 0.02) by 2.2%, 6.5%, and 7.2%, respectively, for CS15. Observed net energy (NE) and the ratio of observed-to-expected NE for maintenance and gain was not influenced (*p* ≥ 0.15) by silage inclusion treatment. Beef production per hectare was not impacted (*p* ≥ 0.13) by corn silage inclusion level. Fecal output was increased, and digestibility coefficients for dry matter, organic matter, and crude protein were decreased in CS30 (*p* ≤ 0.03). Dressing percent and hot carcass weight (HCW) were greater (*p* ≤ 0.02) in CS15. Implant type did not influence any traits measured (*p* ≥ 0.14) except for marbling. Marbling was decreased for PLUS (433 vs. 466 ± 17.5; *p =* 0.02) compared to ONE-F steers. Similar beef produced per hectare of crop land-based upon silage feeding level means producers can feed greater inclusions of corn silage to finishing cattle without impacting carcass quality or beef production; implanting with a coated implant had no detrimental effects to growth performance but increases marbling scores.

## 1. Introduction

Corn silage is a staple feed ingredient among Midwestern cattle producers. The most recently conducted Feedlot Consulting Nutritionist Survey indicated that corn silage was the primary and secondary (37.5% of respondents in both cases) roughage source used in finishing diets [1]. Corn silage production allows farmers to maximize feed tonnage per hectare of land and harvest the crop at an earlier time compared to corn grain. Additionally, corn silage can be harvested at one time to obtain adequate amounts of roughage as compared to other roughage sources, which can require multiple cuttings to obtain equivalent quantities of roughage. This difference in harvest time also allows for flexibility of harvest due to weather conditions, labor availability, and corn market prices [2,3]. However, a long-held belief among cattle producers is that corn silage is best suited for growing cattle and should only be included in finishing rations to maintain optimal ruminal health, mainly as a fiber source. For that reason, many feedlots in the Midwest of the US have replaced it with corn stalks. Most of the prevailing research conducted on corn silage inclusion rates in finishing cattle diets evaluate efficiency on an animal basis (i.e., gain to feed), but few have evaluated corn silage inclusion in terms of beef production from fixed land (i.e., beef production per hectare of cropland). Since land is the limiting factor on production capabilities for most integrated crop-livestock systems, this aspect of efficiency from a fixed land asset base is extremely important for integrated crop-livestock producers. Previous research conducted by this research group suggests that for integrated crop-livestock production systems increased corn silage inclusion in finishing beef diets has no detrimental effect to beef produced per hectare of cropland [4].

Steroidal hormones with anabolic activity have been safely used by the US beef production industry since 1956 [5]. Implants delay fattening, increase frame size, and increase protein accretion, which allows for increased beef production [6]. For over 28 years, anabolic implants containing trenbolone acetate (TBA) and estradiol-17β (E_2_) and modified forms of estradiol such as estradiol benzoate (EB) have been approved for use in confined finishing cattle by the United States Food and Drug Administration (FDA) [5,7]. The FDA has approved coated TBA and estradiol-based steroidal implants for extended hormonal release periods up to 200 d post implantation.

The objective of this experiment was to determine the influence of corn silage inclusion level and terminal implant type (coated or non-coated) containing equal hormonal doses has on animal growth performance, apparent total tract digestibility, beef production per hectare of cropland, and carcass characteristics in finishing steers harvested at a common rib fat endpoint.

## 2. Materials and Methods

Animal care and handling procedures used in this study were approved by the South Dakota State University Animal Care and Use Committee (Approval Number: 19-026E).

### 2.1. Animal Management, Dietary and Implant Treatments

A total of 156 Maine-Anjou × Angus beef steers (initial body weight (BW) 366 ± 37.2 kg) were used to evaluate the effects of increased inclusion rates of corn silage and the effects of coated or non-coated steroidal implants on growth performance, dietary net energy (NE) utilization, apparent total tract digestibility, beef produced per hectare, and carcass traits. Animals were selected from an original pool of 199 steers based upon uniformity. These steers were procured from an unrelated receiving and growing phase study conducted at the Ruminant Nutrition Center (RNC) in Brookings, SD. Approximately 90 d prior to the initiation of the present experiment all steers were boostered for viral respiratory pathogens, clostridia species, and treated for internal and external parasites. Steers were housed in a 7.62 m × 7.62 m concrete surface pen with seven to eight steers per pen. Steers were individually weighed (scale readability of 0.454 kg) on two consecutive days and blocked by BW grouping (*n* = 5 BW blocks). Once assigned to block, steers were assigned to dietary treatment and then implant type. Treatment diets were: (1) 15% (CS15) or (2) 30% dry matter (DM) inclusion of corn silage (CS30). Implant treatments, administered on d 1, were: (1) Synovex PLUS (non-coated implant; 200 mg TBA and 28 mg EB; Zoetis, Parsippany, NJ, USA; PLUS) or (2) Synovex ONE Feedlot (coated implant; 200 mg TBA and 28 mg EB; Zoetis; ONE-F). Feed bunks were managed using a slick bunk approach, minimizing feed present in bunks before each morning feeding, and all diets contained (DM basis) 33 mg/kg monensin sodium. Fresh feed was manufactured twice daily in a stationary mixer (2.35 m^3^; scale readability of 0.454 kg) and offered to steers in equal parts at each feeding (07:00 and 14:00 h). Orts were collected, weighed, and dried in a forced air oven at 60 °C for 24 h if feed became out of condition or prior to weigh days if present. Dry matter intake (DMI) was determined by subtracting the dried orts from the total dry matter (DM) delivered to each pen. Actual diet formulation (Table 1) was based upon weekly DM analysis (drying at 60 °C until no weight change was observed) and corresponding feed batching records. After weekly DM, proximate analysis of each ingredient (except for liquid supplement) was conducted weekly according to: DM (method no. 935.29; [8]), N (method no. 968.06; [9]; Rapid Max N Exceed; Elementar; Mt. Laurel, NJ, USA) where crude protein (CP) was determined form N × 6.25, and ash (method no. 942.05; [8]). Tabular ether extract values for all ingredients were used [10]. Percentages of acid detergent fiber (ADF) and neutral detergent fiber (NDF) were assumed to be 3% and 9% for corn, respectively. Analysis of ADF and NDF composition for all other ingredients was conducted as described by [11].

Steers were given a clostridium type A vaccination (Clostridium Perfringens Type A Toxoid for Cattle, Elanco, Indianapolis, IN, USA) and implant retention was checked on d 28. Implant status was checked by a single trained evaluator, abnormal implant rate was 12.2%; abnormalities included abscess (one steer), abscessed out (one steer), hard (one steer), partial (three steers) and soft inflammation (12 steers). Severe abnormalities such as abscess with implant remaining in the ear or abscessed out only occurred in 1.3% of the cattle. All missing implants were re-administered on trial day 28.

### 2.2. Growth Performance Calculations

Steers were individually weighed on d −1, 1, 28, 56, 84, 112 and the final day, 132. All cattle were shipped on d 132 on feed. Live basis cumulative growth performance was based upon the initial and final shrunk BW (4% shrink was applied to account for digestive tract fill) and carcass-adjusted based growth performance was based upon initial shrunk BW and carcass-adjusted final BW (FBW; hot carcass weight (HCW)/0.63). Average daily gain (ADG) was calculated by the difference in BW during the period of interest, divided by the number of days within the period. The gain to feed (G:F) ratio was calculated by ADG/DMI.

### 2.3. Efficiency of Dietary NE Utilization Calculations

Observed dietary NE was calculated using live shrunk-basis growth performance, and from daily energy gain (EG; Mcal/d): EG = ADG^1.097^ × 0.0557W^0.75^, where W is the mean equivalent shrunk BW (kg; median feeding BW × 478/Mature final BW [14]) based upon median feeding weight (average of live basis initial and final shrunk BW). Mature final body weight was the final BW at 28% empty body fat (EBF) [14,15]. Maintenance energy (EM) was calculated using the equation: EM = 0.077 (median feeding BW, kg^0.75^). Dry matter intake is related to energy requirements and dietary NE for maintenance (NEm) according to the following equation: DMI = EG/(0.877NEm − 0.41), and can be resolved for estimation of dietary NEm by means of the following quadratic formula: $x=\frac{-b\pm \sqrt{b^{2}-4ac}}{2c}$, where *x* is the NEm (Mcal/kg), *a* = −0.41EM, *b* = 0.877EM + 0.41DMI + EG, *c* = −0.877DMI [16]. Dietary NE for gain (NEg) was derived from NEm by the following equation: NEg (Mcal/kg) = 0.877NEm − 0.41 [17].

### 2.4. Beef Production per Hectare Calculations

The beef production per hectare of cropland was calculated from actual intake of corn silage and corn grain (dry-rolled- and high-moisture corn) for each pen. Weekly diet compositions and DMI records were used in these calculations. Corn silage yield was assumed to be 45,700 kg/ha and corn grain yield was calculated to be 10,200 kg/ha. Beef production per hectare was calculated as: (final BW−initial BW)/hectare.

### 2.5. Apparent Total Tract Digestibility Sampling and Analysis

Approximately three weeks prior to harvest, apparent total tract digestibility of diet DM, organic matter (OM), and CP were determined using an internal marker ratio technique. Feed samples were collected from the morning and afternoon feedings starting two days prior to fecal collections. Samples were compiled in equal amounts from each feeding to create a single composite sample of feed for each pen. Fecal samples were taken via rectal palpation at 07:30 h and again at 14:30 h on d 2 of feed collection. Feed and fecal samples were dried and ground through a 1-mm sieve after oven drying at 60 °C until no weight change was observed. Acid insoluble ash was used as an internal marker [18]. Digestibility was calculated using the marker ratio equation: 100−100 × (feed marker/fecal marker) × (fecal variable/feed variable). After DM determination (method no. 935.29; [19]), composite samples were analyzed for N (method no. 968.06; [20]) then N was multiplied by 6.25 to determine CP, and placed in a muffle furnace for 12 h at 500 °C for organic matter determination. One pen was removed from the analysis to irregular digestibility coefficients that fell more than three standard deviations away from the overall mean for all parameters.

### 2.6. Carcass Trait Determination

Steers were harvested when visually appraised to 1.02 cm of rib fat (RF). Cattle were transported to Iowa Premium Beef in Tama, IA after 132 d on feed and harvested the following day. Steers were co-mingled at the time of shipping and remained so until 07:00 the morning of harvest. Steers were tracked throughout the harvest facility by trained personnel. Hot carcass weight was recorded at the hot scale during tag transfer procedure. Trained personnel at the packing plant obtained the carcass trait data such as rib eye area (REA), RF, and United States Department of Agriculture (USDA) marbling scores. Dressing percentage (DP) was calculated as: HCW/(Final BW × 0.96). Yield grade was determined using the USDA regression equation [21]. Estimated empty body fat (EBF) percentage and final BW at 28% EBF (AFBW) were calculated from observed carcass traits [22], and proportion of closely trimmed boneless retail cuts from the chuck, loin, rib and round as a percentage of HCW (retail yield, RY; [23]).

### 2.7. Statistical Analysis

Deads and removals were excluded from all statistical analysis. A total of seven steers were removed from the study due to health reason unrelated to treatment. PLUS/15 had one steer removed due to unresolvable diphtheria. ONE-F/15 had two steers removed due to unresolvable pneumonia (one steer) and heart failure (one steer). Three steers were removed from PLUS/30 due to pneumonia (one steer), poor weight gain (one steer), and heart failure (one steer). One steer was removed from ONE-F/30 due to chronic bloat. Growth performance, beef production per hectare, carcass traits, efficiency of dietary NE utilization, and apparent total tract digestibility were all analyzed using analysis of variance as a randomized complete block design using the GLIMMIX procedure in SAS 9.4 (SAS Inst. Inc., Cary, NC, USA) appropriate for a 2 × 2 factorial arrangement of treatments. Categorical data were analyzed as binomial proportions in the GLIMMIX procedure of SAS 9.4. For all analysis, the model included the fixed effects of steroidal implant, corn silage inclusion level, and their interaction; block was considered a random effect. Least square means were generated using the least squares means statement in SAS. Data means were compared using an F-test. An α of 0.05 determined. One pen was removed from the statistical analysis of digestibility due to all values being greater than three standard deviations away from the mean.

## 3. Results and Discussion

### 3.1. Cumulative Growth Performance

There was no interaction of silage inclusion × implant type (*p* ≥ 0.22) for any growth performance measures (Table 2). Silage inclusion level did not influence live-basis final BW, ADG, or G:F (*p* ≥ 0.19). Carcass adjusted final BW, ADG, and G:F were increased (*p* ≤ 0.02) by 2.2%, 6.5%, and 7.2% respectively for CS15 compared to CS30. Discrepancies amongst live- and carcass-adjusted basis growth performance was due to differences in digestive fill and DP that could not be accounted for in common pencil shrink that was applied for live-basis shrunk growth performance. The main effect of terminal implant type did not influence (*p* ≥ 0.54) any live- or carcass-adjusted growth performance parameters. Others have indicated that feeding greater levels of corn silage to finishing steers did not influence growth performance [4]. However, it has been noted that coated versus non-coated implants differentially affect growth performance [24,25].

Tabular ingredient energy values were in close agreement with cattle performance (Table 2). No interaction of silage inclusion × implant type (*p* ≥ 0.85) or the main effects of silage (*p* ≥ 0.15) or implant (*p* ≥ 0.90) were detected for observed dietary NE based upon performance or the ratio of observed-to-expected dietary NE in the present study. This was consistent with what has been reported by others when greater levels of corn silage is fed to finishing steers [4]. While data comparing efficiency of dietary NE utilization between coated and non-coated implants is limited.

### 3.2. Beef Production per Hectare

No interaction of silage inclusion × implant type (*p* ≥ 0.70) or the main effects of silage (*p* ≥ 0.13) or implant (*p* ≥ 0.56) were detected for agronomic returns (live basis or carcass-adjusted basis beef produced per hectare of cropland). Numerical differences in live-basis versus carcass-adjusted basis agronomic returns was likely due to the same reasons listed above related to applying a generic pencil shrink to diets differing in NDF content and harvesting steers at an equal duration of days on feed. This study demonstrated that producers can effectively feed higher levels of corn silage with no detrimental effects to beef produced per hectare, which was similar to [5]. Additionally, implant type used did not influence agronomic returns to a fixed land base.

### 3.3. Apparent Total Tract Digestibility

Apparent total tract digestibility parameters are presented in Table 3. No significant silage inclusion × implant type interaction was detected for any measurements; however, there was a trend (*p* ≥ 0.08). Intake did not differ between silage group (*p* = 0.41) or implant (*p* = 0.16) during the apparent total tract digestibility measurement period. Fecal output was greater 36.9% (*p* = 0.01) in CS30 compared to CS15. Digestibility coefficients for DM, OM, and CP were lesser (*p* ≤ 0.03) with greater silage inclusion, but were not influenced (*p* ≥ 0.20) by steroidal implant type. As forage inclusion increases, fiber increases, and DM digestibility decreases and so does the amount of nutrients found in fecal matter [26]. This may be due to a greater passage rate and increased ruminal fill associated with greater corn silage inclusion.

### 3.4. Carcass Traits

Carcass trait responses are located in Table 4. No interaction of silage inclusion × implant type was detected for any carcass traits (*p* ≥ 0.16). Silage had no effect on REA, RF, USDA marbling score, calculated yield grade, retail yield, estimated EBF, final BW at 28% EBF, or the distribution of USDA Quality or Yield grades. Dressing percentage was greater for CS15 (64.52% vs. 63.47% ± 0.250%; *p* = 0.01) which can be attributed to decreased digestive fill compared to the CS30 diet. With cattle finishing at a similar final body weight (588 vs. 585 ± 8.0 kg; *p* = 0.62) with differing DP it was not surprising HCW was greater in CS15 (379 vs. 371 ± 13.1 kg; *p* = 0.02).

When comparing implant treatments, no differences were observed for dressing percentage, hot carcass weight, ribeye area, or rib fat (*p* ≥ 0.22). Marbling differed between implant treatments (433 to 466 ± 17.5; *p* = 0.02) for PLUS and ONE respectively. This was likely due to alterations of implant type on adipogenic gene expression [27,28], although this was not evaluated in the present study. Others have indicated that marbling is increased in heifers administered a single coated implant or an initial and terminal implant with a non-coated implant [29,30].

## 4. Conclusions

Feeding increased levels of corn silage in finishing diets does not alter live-basis growth performance, however, carcass-adjusted growth performance is decreased. Depending upon marketing options (live or dressed basis) these differing responses should be exploited. When marketing on a HCW basis, using a lower level of corn silage in the finishing phase can result in heavier HCW when cattle are harvested at equal days on feed. Agronomic returns per hectare did not differ due to silage inclusion level suggesting that integrated crop-livestock systems can harvest and feed more corn silage without detriment to returns to a fixed land base. Terminal implant type (coated vs. non-coated) did not influence growth performance or carcass characteristics other than marbling scores. Use of these differing technologies in practice should be determined upon the method in which the beef cattle are marketed, cost of the implant, and the improvements in revenue for cattle that are rewarded a premium for greater quality grades.

## Figures and Tables

**Table 1:** Actual diet formulation and composition based upon weekly DM and nutrient composition determinations for 15% corn silage (CS15) and 30% corn silage (CS30) ^1,2^.

	d 1 to 98				d 99 to 132			
Item	CS15	(sd) ^3^	CS30	(sd)	CS15	(sd)	CS30	(sd)
Samples, *n*	15		15	-	5	-	5	-
High moisture corn, %	36.03	(0.287)	28.50	(0.314)	-	-	-	-
Dry rolled corn, %	36.61	(0.346)	28.97	(0.397)	73.00	(0.230)	57.87	(0.295)
Corn silage (CS), %	15.34	(0.445)	30.55	(0.729)	15.24	(0.171)	30.40	(0.277)
Suspension supplement ^4^, %	5.02	(0.052)	5.00	(0.072)	4.90	(0.065)	4.89	(0.063)
Pelleted supplement ^5^, %	7.00	(0.063)	6.98	(0.093)	6.86	(0.079)	6.84	(0.075)
Dry matter, %	64.32	(0.667)	54.56	(0.783)	69.59	(0.921)	57.82	(0.752)
Crude protein, %	12.32	(0.459)	12.07	(0.456)	11.85	(0.265)	11.49	(0.298)
Neutral Detergent Fiber, %	13.57	(0.599)	18.53	(1.194)	14.18	(0.402)	19.74	(0.785)
Acid Detergent Fiber, %	6.12	(0.249)	9.20	(0.484)	6.20	(0.176)	9.37	(0.358)
Ash, %	4.87	(0.115)	5.34	(0.150)	4.83	(0.194)	5.29	(0.254)
NEm ^6^, Mcal/kg	2.08	(0.002)	2.01	(0.003)	2.05	(0.001)	1.96	(0.002)
NEg ^6^, Mcal/kg	1.40	(0.002)	1.33	(0.003)	1.38	(0.001)	1.31	(0.001)

^1^ All values except for dry matter (DM) on a DM basis. ^2^ calculated from weekly ingredient assays and feed batching records. ^3^ sd = standard deviation. ^4^ Provided micronutrients to meet or exceed [12] requirements and provided 33 mg/kg monensin sodium. ^5^ Contains (DM basis): 85.70% soybean meal, 2.85% trace mineralized salt, 2.85% urea, and 8.60% dry rolled corn. ^6^ Based upon tabular NE values for ingredients for maintenance (NEm) and gain (NEg) [13].

**Table 2:** Cumulative live (shrunk) and carcass-adjusted (hot carcass weight (HCW)/0.63) growth performance responses and beef production per hectare of cropland in finishing diets containing 15% or 30% (DM basis) corn silage and administration of a non-coated (PLUS) or coated (ONE-F) implant containing 200 mg of trenbolone acetate and 28 mg of estradiol benzoate ^1^.

	15% Corn Silage (CS15)		30% Corn Silage (CS30)			*p*-Value		
Item	PLUS	ONE-F	PLUS	ONE-F	SEM	Silage (S)	Implant (I)	S × I
Pens, *n*	5	5	5	5	-	-	-	-
Steers, *n*	38	37	36	38	-	-	-	-
Live basis ^2^								
Initial body weight (BW), kg	370	369	368	368	-	-	-	-
Final BW, kg	589	586	582	587	8.0	0.62	0.86	0.51
Average daily gain (ADG), kg	1.70	1.65	1.62	1.66	0.054	0.46	0.89	0.22
Dry matter intake (DMI), kg	10.10	9.92	10.08	10.21	0.169	0.29	0.85	0.22
Gain to feed ratio (G:F)	0.168	0.166	0.161	0.163	0.005	0.19	1.00	0.60
Carcass-adjusted basis ^3^								
BW, kg	603	601	589	590	6.622	0.02	0.86	0.70
ADG, kg	1.81	1.75	1.67	1.68	0.044	0.01	0.54	0.30
G:F	0.179	0.177	0.166	0.165	0.006	0.01	0.61	0.89
Observed dietary NE ^4^, Mcal/kg								
Maintenance	2.05	2.05	2.02	2.02	0.051	0.43	0.94	0.94
Gain	1.39	1.39	1.36	1.36	0.045	0.43	0.94	0.94
Observed to expected dietary NE ^5^								
Maintenance	0.99	0.99	1.02	1.02	0.025	0.15	0.91	0.87
Gain	0.99	0.99	1.03	1.03	0.032	0.23	0.90	0.85
Agronomic return								
Live basis beef produced, kg/hectare	2027.0	2011.0	2087.0	2109.0	70.7	0.13	0.96	0.70
Carcass-adjusted beef produced, kg/hectare	2159.0	2137.0	2146.0	2131.0	42.3	0.76	0.56	0.92

^1^ Deads and removals excluded. ^2^ A 4% shrink was applied to all BW measures in order to account for gastrointestinal tract fill. ^3^ Calculated from HCW/0.63. ^4^ Based upon live growth performance. ^5^ Actual diet NE based upon tabular values and diet formulation were: 2.06 Mcal/kg of NEm and 1.40 Mcal/kg of NEg for CS15; 1.98 Mcal/kg of NEm and 1.32 Mcal/kg of NEg.

**Table 3:** Digestibility of dry matter, organic matter, and crude protein in finishing diets containing 15% or 30% (DM basis) corn silage and administration of a non-coated (PLUS) or coated (ONE-F) implant containing 200 mg of trenbolone acetate and 28 mg of estradiol benzoate ^1^.

	15% Corn Silage		30% Corn Silage			*p*-Values		
Item	PLUS	ONE-F	PLUS	ONE-F	SEM ^2^	Silage (S)	Implant (I)	S × I
*n*, Pens	5	5	4	5	-	-	-	-
DMI, kg	11.89	11.64	12.17	11.74	0.329	0.41	0.16	0.71
Fecal Output, kg	2.92	3.02	4.52	3.61	0.50	0.01	0.15	0.08
Nutrient digestibility, %								
Dry Matter	75.19	74.12	60.57	69.39	3.130	0.01	0.21	0.09
Organic Matter	76.95	75.86	64.96	71.30	2.923	0.01	0.20	0.08
Crude Protein	67.37	61.95	49.42	58.75	6.447	0.03	0.66	0.12

^1^ Acid insoluble ash was used as an internal marker. Using the marker ratio technique to estimate digestibility coefficients. ^2^ SEM: standard error of the mean.

**Table 4:** Carcass trait responses in finishing diets containing 15% or 30% (DM basis) corn silage and administration of a non-coated (PLUS) or coated (ONE-F) implant containing 200 mg of trenbolone acetate and 28 mg of estradiol benzoate.

	15% Corn Silage		30% Corn Silage			*p*-Value		
Item	PLUS	ONE-F	PLUS	ONE-F	SEM	Silage (S)	Implant (I)	S × I
Pens, *n*	5	5	5	5	-	-	-	-
Steers, *n*	38	37	36	38	-	-	-	-
Dress ^1^, %	64.56	64.48	63.69	63.25	0.501	0.01	0.48	0.62
Hot carcass weight (HCW), kg	380	378	371	372	4.17	0.02	0.86	0.70
Ribeye area, cm^2^	93.35	92.97	92.45	91.87	1.142	0.24	0.55	0.93
Rib fat, cm	1.14	1.07	1.12	0.99	0.112	0.53	0.22	0.71
Marbling score ^2^	436	451	429	480	17.5	0.42	0.02	0.16
Yield Grade	2.67	2.61	2.62	2.52	0.139	0.50	0.43	0.87
Retail yield ^3^, %	50.75	50.88	50.86	51.04	0.279	0.50	0.45	0.88
Estimated empty body fat (EBF) ^4^, %	28.54	28.32	28.26	28.12	0.676	0.63	0.71	0.93
Final BW at 28% EBF ^4^, kg	589	590	580	583	8.9	0.23	0.74	0.87
Select, %	31.43	19.64	34.28	19.64	8.459	0.87	0.14	0.87
Choice, %	63.21	70.00	57.03	63.57	8.369	0.46	0.44	0.99
Upper two-thirds Choice, %	5.36	10.36	8.69	8.58	3.827	0.84	0.53	0.51
Prime, %	0.00	0.00	0.00	8.21	2.812	0.16	0.16	0.16
Yield Grade 1, %	10.71	16.78	9.17	13.93	5.303	0.68	0.62	0.90
Yield Grade 2, %	62.86	45.36	55.95	42.14	11.956	0.68	0.21	0.88
Yield Grade 3, %	26.43	37.86	34.88	43.93	11.479	0.54	0.39	0.92

^1^ Calculated as HCW/final BW (shrunk 4%). ^2^ 400 = small^00^ (United States Department of Agriculture (USDA) Low Choice). ^3^ Retail yield of the round, loin, rib, and chuck as a percentage of HCW. ^4^ Calculated according to the equations described by: Guiroy et al. 2001 [14].

## Data Availability

Data available upon request.
